# Regenerative Wound Dressings for Skin Cancer

**DOI:** 10.3390/cancers12102954

**Published:** 2020-10-13

**Authors:** Teodor Iulian Pavel, Cristina Chircov, Marius Rădulescu, Alexandru Mihai Grumezescu

**Affiliations:** 1 Department of Science and Engineering of Oxide Materials and Nanomaterials, Faculty of Applied Chemistry and Materials Science, University Politehnica of Bucharest, RO-060042 Bucharest, Romania; teodor_iulian.pavel@stud.fils.upb.ro (T.I.P.); cristina.chircov@upb.ro (C.C.); agrumezescu@upb.ro (A.M.G.); 2 Department of Inorganic Chemistry, Physical Chemistry and Electrochemistry, Faculty of Applied Chemistry and Materials Science, University Politehnica of Bucharest, 1–7 Polizu Street, 011061 Bucharest, Romania

**Keywords:** wound dressings, skin cancer, tumor excision, tumor recurrence, natural biocompounds, anti-cancer agents, bioactive wound dressing

## Abstract

**Simple Summary:**

As the currently and commonly applied treatment strategies for skin cancer are highly invasive and possibly disfiguring, new approaches should focus on developing wound dressings that could promote both tumor eradication and skin regeneration. In this context, we aim to provide a complete overview on the limitations of currently available topical field treatments and to emphasize on the potential of natural biocompounds with anti-cancer and anti-microbial effects that could be introduced into wound dressings consisting of biopolymers with regenerative capacities. This paper could represent the first step towards the scientific advancement of regenerative wound dressings for skin cancer therapy.

**Abstract:**

Skin cancer is considered the most prevalent cancer type globally, with a continuously increasing prevalence and mortality growth rate. Additionally, the high risk of recurrence makes skin cancer treatment among the most expensive of all cancers, with average costs estimated to double within 5 years. Although tumor excision is the most effective approach among the available strategies, surgical interventions could be disfiguring, requiring additional skin grafts for covering the defects. In this context, post-surgery management should involve the application of wound dressings for promoting skin regeneration and preventing tumor recurrence and microbial infections, which still represents a considerable clinical challenge. Therefore, this paper aims to provide an up-to-date overview regarding the current status of regenerative wound dressings for skin cancer therapy. Specifically, the recent discoveries in natural biocompounds as anti-cancer agents for skin cancer treatment and the most intensively studied biomaterials for bioactive wound dressing development will be described.

## 1. Introduction

Among the noncommunicable diseases, which account for 71% of global deaths, cancer is the second leading one, with 18.1 million cases and 9.6 million deaths worldwide in 2018. According to the World Health Organization (WHO), the numbers will double by 2040, with the highest increase in low- and middle-income countries [1,2,3]. Cancer is characterized by an abnormal growth of cells, which further invade and spread throughout different body organs through metastasis [4,5,6]. Its metabolism is highly complex, depending on a series of factors, including genetic and epigenetic alterations, the surrounding environment, the tissue of origin, and the systemic host metabolism [7]. Consequently, cancer cells possess a remarkable ability of surviving and adapting to various stress conditions, such as oxidative and metabolic stress, hypoxia, and nutrient deprivation [8].

Skin cancer is considered the most prevalent cancer type globally and in the United States, with a continuously increasing prevalence and mortality growth rate [9,10,11,12]. Skin cancer is characterized by an imbalance in cell homeostasis and excessive cell proliferation as a result of cancer-associated gene mutations, such as skin proto-oncogenes and tumor suppressors within skin cells [12]. Depending on the type of cells affected, there are two major types of skin cancer, namely non-melanoma and cutaneous melanoma [12]. On one hand, non-melanoma skin cancers, predominantly comprising basal cell carcinoma and cutaneous squamous cell carcinoma, originate from the keratinocytes within the epidermis and account for approximately five million new cases and 65,000 associated deaths yearly [10,11,12,13]. Other types of non-melanoma skin cancers include Merkel cell carcinoma, Kaposi sarcoma, dermatofibrosarcoma protuberans, primary cutaneous B-cell lymphoma, sebaceous carcinoma, and atypical fibroxanthoma, which are significantly rarer [13,14]. On the other hand, melanoma originates from melanocytes within the deepest layer of the epidermis and, although its prevalence is considerably lower, it has the worst prognosis, with 280,000 new cases and 60,000 associated deaths reported yearly [10,11,12,13]. Moreover, the incidence of skin cancers is continuously increasing, which could be associated with higher UV radiation exposure [11,15].

Reducing cancer-related mortality rates has become a major challenge faced by societies, governments, and medical and scientific communities [16]. However, conventional treatment options, including chemotherapy, radiotherapy, immunotherapy, and gene and hormone therapy, are associated with various drawbacks that limit their efficiency [4]. In this regard, cancer treatment generally involves a combination of therapies in order to control the evolution of the disease [17]. Nonetheless, chemotherapy is still considered the most efficient strategy and it is widely used in most cases, with more than 200 anti-cancer drugs developed that include cytostatics, anti-hormonal drugs, recombinant proteins and antibodies for molecular targeted therapy, and supportive care drugs [16,17,18]. In the case of non-melanoma skin cancer, radical tumor excision remains the most effective approach among the available strategies [14,19,20]. However, radical excision may not be possible due to patient co-morbidities or unfavorable cosmetic defects, and non-surgical approaches, such as cryotherapy, curettage, electrodessication, topical therapy, photodynamic therapy, or radiotherapy become the only option [13,14,19,20]. By contrast, treatment of melanoma involves surgery followed by radiotherapy, immunotherapy, and chemotherapy [13,20]. Nevertheless, there is a high recurrence risk in skin cancer therapy, which makes its treatment among the most expensive of all cancers, with average costs estimated to double within 5 years [9,12,20]. Additionally, surgical interventions could be disfiguring, requiring additional skin grafts for covering the defects [20].

Generally, the skin plays four fundamental roles, namely body protection against physical, chemical, and bacteriological damages, thermoregulation through skin vasculature and eccrine sweat glands, prevention of dehydration, and conduction of neurosensory information which further contributes to endocrine function and immune surveillance regulations [21,22]. Hence, maintaining its integrity is fundamental. In this context, post-surgery management should involve the application of wound dressings for promoting skin regeneration and preventing tumor recurrence and microbial infections, which still represents a considerable clinical challenge [23]. Such wound dressings should maintain a moist environment and allow for fluid exchange, which would promote wound healing and regeneration [24], and provide a controlled release of bioactive compounds for anti-cancer and anti-microbial purposes [25].

Therefore, the aim of this paper is to provide an up-to-date overview regarding the current status of regenerative wound dressings for skin cancer therapy. Specifically, the recent discoveries in natural biocompounds as anti-cancer agents for skin cancer treatment and the most intensively studied biomaterials for bioactive wound dressing development will be described.

## 2. Topical Field Treatments

Generally, topical field treatments used for skin cancer therapies involve the application of creams or gels containing imiquimod, 5-fluorouracil, ingenol mebutate, or diclofenac [26,27], with regimens lasting from 2 to 90 days, depending on the tumor complexity and dosage. However, such prolonged and complex treatments are usually associated with non-adherence and non-persistence to the prescribed treatment [28]. Additionally, these agents are chemically and pharmacologically different, with various related side effects (Table 1) [29].

### 2.1. Imiquimod

**Figure 1:**
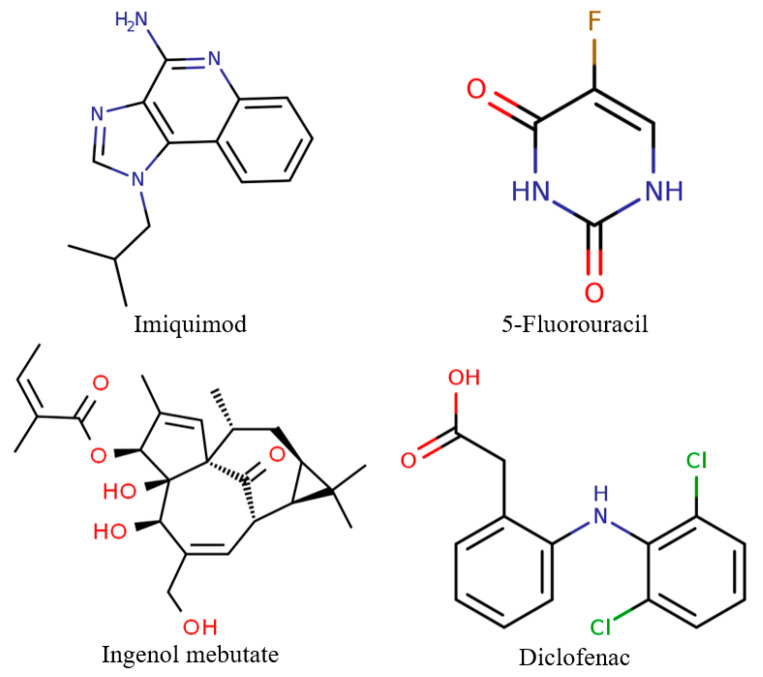
The chemical structures of the four agents commonly used for topical field treatment of skin cancers.

Imiquimod (1-isobutyl-1*H*-imidazo(4,5-c)quinolin-4-amine, Figure 1), is a low molecular weight nucleoside analog of the imidazoquinoline family with immunomodulating properties and indirect anti-viral and anti-tumor effects [30,31,32,33,34,35,36]. Additionally, it acts as a potent antagonist for the toll-like receptors 7 and 8 [30,31,35,37]. Initially, imiquimod was used for the treatment of human papilloma virus-associated genital and perianal warts. Presently, it is commonly applied for treating actinic keratosis and superficial basal cell carcinoma, with no sufficient information to prove its efficiency for nodular basal cell carcinoma or squamous cell carcinoma [30,31,32,34,36].

The anti-tumor effects of imiquimod, although not completely understood, could be attributed to two major underlying mechanisms. On one hand, it activates the dendritic cells and monocytes where the toll-like receptors are predominantly expressed, which leads to the NF-κB-dependent secretion of pro-inflammatory cytokines and chemokines, such as interferon-α, tumor necrosis factor-α, and interleukin-6 and -8. Consequently, antigen-presenting cells and innate immunity components are activated, which will generate profound T-helper 1-mediated anti-tumor immune responses [30,31,32,33,34,35]. On the other hand, imiquimod facilitates pro-inflammatory activities by interfering with adenosine receptor signaling pathways [30].

Imiquimod for topical administration is used as 3.75% or 5% creams and treatment involves 5 to 7 times per week applications, once a day, for 6 to 12 weeks [31]. In this manner, it provides a non-surgical, non-invasive, self-administered therapeutic option with relatively low costs [35,38]. For 6-week treatments using 5% creams, clearance rates are between 52% and 81% [31,32]. However, imiquimod treatments have been associated with a variety of side effects, including erosion, ulceration, healing with scarring or hyperpigmentation, erythema, vesiculation, edema, weeping, pruritus, scaling, crusting, burning, and pain [31,32,33,34]. Additionally, it was observed that higher clearance rates lead to higher inflammatory reactions [31,32]. Moreover, imiquimod is characterized by an inability to permeate through the dermis layer due to its low water solubility and the interactions between the amine groups and the aninonic components of the skin [36,38].

### 2.2. 5-Fluorouracil

5-Fluorouracil (5-fluoro-1*H*-pyrimidine-2,4-dione, Figure 1), is a pyrimidine analogue [31,39,40,41] which belongs to the class of anti-metabolite drugs [31,35,41,42,43]. As a chemotherapeutic agent, it has been widely applied in the treatment of malignant diseases [42]. Moreover, it has been used for the topical treatment of superficial basal cell carcinoma and, while it is not recommended for nodular or infiltrative basal cell carcinoma, it is used in very old patients with no other therapeutic options [27,31,42,43].

The main underlying mechanism for its anti-tumor character involves blocking the conversion of deoxyuridine into thymidine as a consequence to the irreversible binding to thymidylate synthase through the cofactor 5,10-methylenetetrahydrofolate. In this manner, DNA synthesis in cancer cells is inhibited, which results in reduced cell proliferation and increased apoptosis [35,41,43]. 5-Fluorouracil metabolism depends on its degradation by the dihydropyrimidine dehydrogenase enzyme which reduces the molecule to its inactive form, dihydrofluorouracil [44].

For topical applications, 5-fluorouracil is available as 2% and 5% solutions or 5% cream and it is used twice a day for 2 or more weeks. For superficial basal cell carcinoma treatment, clearance rate is 90% and for squamous cell carcinoma in situ, between 48% to 85% [31,43]. Its advantage relies on the possibility of the patient to apply it at home, but it can be challenging for elderly individuals who might need assistance [31,43]. Common side effects include allergic contact and irritant dermatitis, erythema, pruritus, erosions, hyper- or hypopigmentation, and pain [31,43,44]. In this case, there is no correlation between clearance rates and adverse reactions [31].

### 2.3. Ingenol Mebutate

Ingenol mebutate, also known as ingenol-3-angelate (Figure 1), is a diterpene ester derived from the Euphorbia peplus plant species [45]. Known for its anti-cancer properties, ingenol mebutate is a novel therapeutic agent used for the treatment of skin conditions, including actinic keratosis, verrucae, and superficial basal cell carcinoma [31,45,46].

There is a dual underlying mechanism that could be attributed to its anti-cancer potential, consisting of mitochondrial destruction induced by increased intracellular calcium levels and subsequent epidermal cell death through necrosis and apoptosis. The following stage involves protein kinase C activation, which further stimulates infiltration of neutrophils, production of pro-inflammatory cytokines, expression of endothelial adhesion molecules, and formation of tumor-specific antibody, leading to a neutrophil-mediated antibody-dependent cellular cytotoxicity [31,35,45,46,47]. In this manner, inflammation manifests within hours of application through erythema and edema and subsequent pustules, epidermal flaking, and crusting, which subside in less than 2 weeks [47].

Ingenol mebutate is available in 0.015% and 0.05% gels and it is administered for 2 to 7 consecutive days, with clearance occurring in approximately 63% of patients [31,46,47]. While its application is advantageous due to self-administration and short application periods, it is usually associated with adverse events, including erythema, edema, pruritus, and pain [31,47,48].

### 2.4. Diclofenac

Diclofenac (2-(2-(2,6-dichlorophenylamino)phenyl)acetic acid, Figure 1), also known for its sodium salt sold as Voltaren, is a non-steroidal anti-inflammatory drug widely used for actinic keratosis treatment and can be an adjuvant for basal cell carcinoma, squamous cell carcinoma, and melanoma skin metastases therapy [35,49,50].

Its mechanism of action, although not completely elucidated, could involve the inhibition of cyclooxygenase-1 and -2, enzymes involved in reducing prostaglandin formation from arachidonic acid, which reduces PGE2 synthesis and dysplastic keratinocytes in cancerous lesions [31,35,50,51]. Additionally, it might interfere with the SHH and Wnt signaling and lead to cancer cell apoptosis and inhibit angiogenesis and proliferation [31,35,50].

The current formulation contains 3% diclofenac in 2.5% hyaluronic acid which is applied twice daily for 8 weeks. Clearance rates range between 38% to 47% for actinic keratosis and 64.3% for superficial basal cell carcinoma [31,35,51]. Although it can be self-applied, there are frequent side effects associated, including erythema, erosion, allergic contact dermatitis, photoallergy, and pruritus [31].

## 3. Natural Anti-Cancer Agents for Skin Cancer

Oxidation by solar radiation has become a principal cause for the development of skin diseases through the excessive production of reactive oxygen species which consequently leads to inflammation and DNA and protein damages [52,53]. Recent studies have reported a direct causal connection between inflammation and cancer development. In this context, cancer-related inflammation occurs through two main pathways, namely the intrinsic pathway produced by genetic events as a causing factor of inflammation and neoplastic transformation and the extrinsic pathway through which carcinogenesis is promoted by inflammatory conditions [54].

In this regard, plants are important sources which produce secondary metabolites for protection purposes. Specifically, such compounds exhibit DNA protection, antioxidant, anti-inflammatory, chemopreventive, and chemotherapeutic activities. Among them, flavonoids, phenolic acids, lignins, stilbenes, and retinoids, are the most commonly studied for their anti-cancer potential, especially for skin cancers [52,53].

### 3.1. Flavonoids

Flavonoids are a class of antioxidants biosynthesized via the shikimic acid pathway from acetic acids or phenylalanine derivatives [55]. Although they can be divided into flavones, flavonols, isoflavonoids, isoflavones, flavanones, flavanols, and anthocyanidins, most of the bioactive compounds belong to the former three groups, which have received great scientific interest in the past years [56,57,58]. There is an increasing number of studies reporting the anti-cancer or cancer preventive effects of flavonoids against prostate, colorectal, breast, thyroid, lung, ovarian, and skin cancers [59,60].

The chemopreventive character of flavonoids relies on their potential to inhibit new cancer cell development, prevent carcinogens from reaching activation sites, decrease compound toxicity by inhibiting their metabolism [59]. The molecular mechanisms responsible involve apoptosis induction, cell cycle arrest by inhibiting key regulators, metabolizing enzymes inhibition and subsequent inactivation of carcinogenic compounds, reactive oxygen species scavenging, angiogenesis inhibition, DNA repair mechanism initiation, and cancer cell proliferation and invasiveness suppression [56,59,60,61]. Additionally, some flavonoid compounds have shown to prevent cancer relapse and chemotherapy failure by considerably inhibiting multidrug resistance [59].

In skin cancers, flavonoids have shown anti-inflammatory, anti-proliferative, anti-angiogenic, and apoptotic activities. Specifically, topical administration of flavonoids leads to skin absorption and consequent activation of a cascade of protective signaling pathways and cell cycle arrest in G0-G1 and G2-M phases [60]. Additionally, dietary intake of flavonoid-rich products has shown to ensure DNA protection of skin cells exposed to carcinogenic factors, such as UV radiation [56]. Among flavonoid compounds, apigenin, quercetin, silymarin, diosmetin, genistein, fisetin, and luteolin, have been identified as potential anti-cancer agents in skin cancers (Table 2) [57,59].

### 3.2. Phenolic Acids

Phenolic acids are a class of antioxidant compounds which are formed through the substitution of hydrogen atoms present on the benzene rings by a carboxylic and at least one hydroxyl group. They are ubiquitously present in plants and human metabolites and, by contrast to flavonoids, have suitable water solubility and high bioavailability [87]. Phenolic acids exhibit their anti-cancer properties by inducing apoptosis through the ASK-1, caspase-3, JNK-p38, and pRb pathways, suppressing cell cycle by p21, bcl2, and bcl-x upregulation and bim, bax, puma ans noxa downregulation, and reducing proliferation and angiogenesis by EGFR, MAPK, mTOR, PI3K/Akt, FAK/PTK2, and JAK/STAT upregulation. Additionally, they are involved in modulation of inflammatory and cytokine genes expression, which are further implicated in cellular proliferation, invasion, and metastasis inhibition [12]. Furthermore, as they are highly potent antioxidants owing to the presence of the hydroxyl substituent on the benzene ring, phenolic acids can act as chemopreventive agents for UV-induced skin cancer [88].

Among them, syringic acid, ferulic acid, and caffeic acid have received a considerable interest in the recent years (Table 3). Specifically, studies have shown that treatment with syringic acid led to suppressed UV-induced cyclooxygenase-2, matrix metalloproteinase-1, and prostaglandin E2 expression and activator protein-1 activity [89]. Additionally, it inhibited the Nox/PTP-κ/EGFR pathway and subsequently reactive oxygen species formation. Its anti-cancer character might be attributed to the presence of the methoxy groups on the benzene ring [90,91]. Similarly, ferulic acid has shown to exhibit photoprotective character which could prevent UV-induced carcinogenesis. The antioxidant properties are generally based on its structural features, namely the hydroxyl group which neutralizes the reactive oxygen species as it acts as an electron donor, the vinyl chain and the methoxyl group which increase molecule stability, and the carboxylic group which prevents lipid peroxidation [92]. Moreover, caffeic acid acts as a chemopreventive agent by modulating inflammatory signaling [93], suppressing the rapamycin cascade signaling, inducing apoptosis [94], and altering cell cycle and caspase gene expression [95].

### 3.3. Lignin

**Figure 2:**
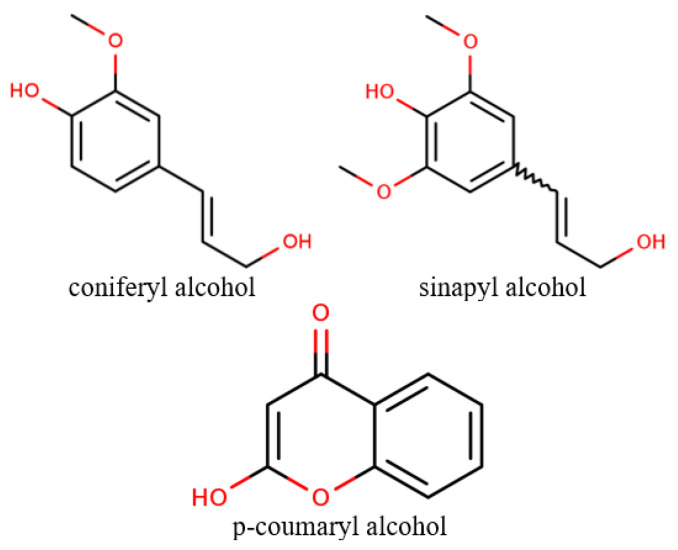
The chemical structures of the common monolignol precursors of lignin.

Lignin is the second most abundant renewable resource on earth, comprising a three-dimensional heterogenous and phenolic polymer network synthesized in the cell wall of higher plants. The term involves a variety of natural aromatic compounds obtained through the oxidative coupling of monomeric precursors [99,100]. The main monomers implicated in lignin structure, also known as monolignols, are coniferyl alcohol, sinapyl alcohol, and *p*-coumaryl alcohol (Figure 2) [100,101]. Moreover, lignin structures is highly influenced by the extraction processes and the presence of different functional moieties, such as hydroxyl, methoxyl, carbonyl, and carboxyl groups. Through hydroxyalkylation reaction processes, such as phenolation, demethylation, or methylolation, lignin is transformed to phenolic compounds [101].

Although it has not yet been converted into high-value products at large scales, lignin has shown to exhibit promising functions, including antioxidant, anti-microbial, and UV blocking. Additionally, as they do not cause cytotoxicity, lignin-based products could be applied for biomedical purposes [99]. In this context, lignin is a widely used bio-based UV-blocking material owing to its UV-absorbing phydroxyphenyl, guaiacyl, and syringyl phenylpropanoid units [99,102].

### 3.4. Stilbenes

Stilbenes, a class of non-flavonoid phenolic compounds, generally consist of C_6_-C_2_-C_6_ structures with two hydroxyl groups on the A ring and one on the B ring and are regarded as 1,2-diphenylethylenes [103,104,105,106]. Most stilbenes are found in plants as aglycones or glycosides, thus offering anti-fungal and anti-bacterial protection [105,106]. Additionally, studies have demonstrated strong antioxidant and chemopreventive properties of the stilbenoid group [103]. Most common stilbene examples are the phytoalexins resveratrol and the resveratrol metabolites, pterostilbene and piceatannol (Table 4) [103,105].

Owing to its promising anti-inflammatory, antioxidant, anti-cancer, anti-mutagenic, anti-aging, and anti-allergenic properties, resveratrol is one of the most intensively studied stilbenes. Initially identified as an SIRT1 activator which regulates energy homeostasis and mitochondrial biogenesis within cells, it is now studied for its apoptosis promoting capacity by enhancing sensitivity to tumor necrosis factor-α and suppressing NF-κB activation [104,106,107]. In skin cancer applications, studies have shown that topical administration of resveratrol not only improved skin elasticity, hydration, and luminosity [108], but also provided protection against UV radiations and UV-induced carcinogenesis by regulating protein activity regarding apoptosis, decreasing reactive oxygen species production, inhibiting tumor incidence and tumorigenesis, and modulating cell cycle molecules and cell signaling pathways [109]. Additionally, it has proved its potential to initiate senescence in squamous cells carcinoma cells through its autolysosome form blockade and Rictor protein expression downregulation, which altered cancer cell skeleton and suppressed cancer progression [110].

Pterostilbene and piceatannol, two resveratrol analogs, have also proved anti-cancer activities similar or superior to resveratrol levels [104]. On one hand, pterostilbene, which has a higher lipophilicity and, consequently higher bioavailability and membrane permeability, possesses intrinsic antioxidant properties by activating the nuclear factor erythroid 2-related factor 2 and anti-inflammatory character by targeting inducible nitric oxide synthase, cyclooxygenases, leukotrienes, NF-κB, tumor necrosis factor-α, and interleukins [111]. Moreover, it can also increase lysosome size and induce membrane destabilization and caspase-independent cell death [112]. On the other hand, piceatannol leads to Bax upregulation, Bcl-2 downregulation, and caspase-3 activation, which induced melanoma cell apoptosis [114].

### 3.5. Retinoids

**Figure 3:**
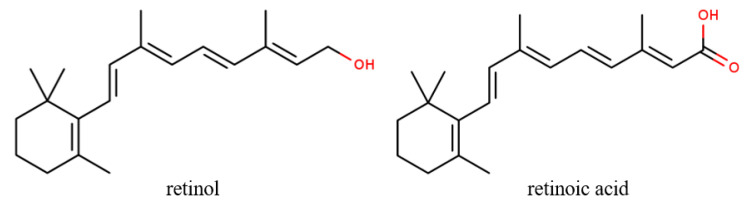
The chemical structures of retinol and retinoic acid, the most common retinoids topically applied for skin cancer.

Retinoids are polyphenolic compounds derived from vitamin A. Among its derivatives, retinol and retinoic acid (Figure 3), also known as tretinoin, are the most commonly used for oral or topical administration to prevent skin cancer [21]. Mechanisms that could be attributed to its anti-tumor effects are based on inhibiting UV-induced phosphorylation of ERK1/2, JNK, and p38 proteins of the MAPK family [12]. Furthermore, oral administration has also proved efficient against basal cell carcinoma, squamous cell carcinoma, and actinic keratosis [115].

## 4. Wound Dressings in Skin Cancer

**Figure 4:**
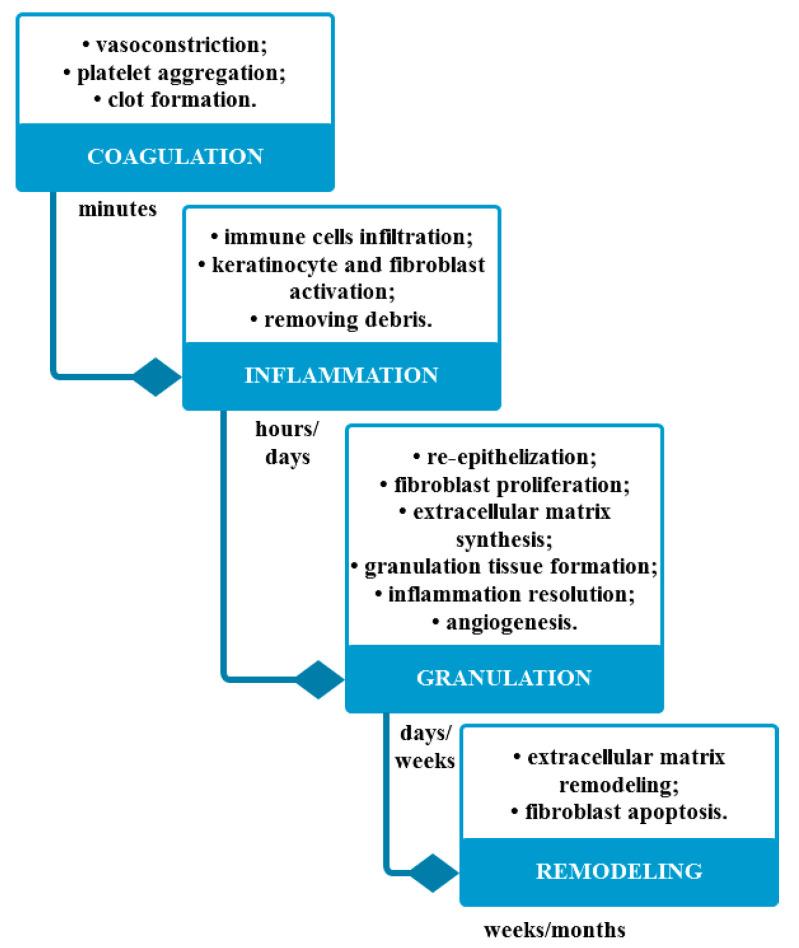
The four main stages involved in the cutaneous wound healing processes and the implicated mechanisms.

The skin has the ability to repair itself following injuries due to surgery, trauma, or burns through cutaneous wound healing processes [116]. Generally, it involves four main stages, namely coagulation, inflammation, granulation, and remodeling, finally resulting in wound closure (Figure 4) [116,117]. However, wound areas larger than 2 cm^2^, wound duration longer that 2 months, and increased wound depth which results in tendon, ligament, or bone exposure are the three main factors which delay or stop the healing process. In this context, research is focused on developing wound dressings not only for protection purposes, but also to promote healing and regeneration processes [118,119]. Therefore, the application of healing-promoting approaches has shown to trigger, accelerate, and enhance wound healing, re-epithelialization, and collagen formation, which subsequently results in reduced scar formation and complications [120,121].

Based on their interaction with the wound site, wound dressings can be divided into three main groups, namely inert or passive, interactive, and bioactive. As inert or passive dressings are ordinary materials designed only for covering and providing protection to the wound against pathogen contamination from the external environment [122], they are not suitable for regenerative applications in skin cancer. On the contrary, interactive wound dressings are capable of altering the wound microenvironment, by interacting with the surface and promoting healing processes [123]. Moreover, interactive dressings support all the stages involved in the healing process, such as debris removal, granulation tissue formation, and re-epithelialization, while also decreasing exudate formation and preventing bacterial colonization. Among them, hyaluronic acid, collagen, and alginate-based dressings are the most commonly and extensively investigated products [25,118]. These natural polymers are highly advantageous owing to their cell proliferation and growth, tissue regeneration, non-toxicity, minimal inflammatory and immunological response induction characteristics. Additionally, their chemical structures provide unique physicochemical properties which are fundamental for skin regeneration processes [124].

Hyaluronic acid is a glycosaminoglycan and one of the most important extracellular matrix components ubiquitously found in the connective tissues of all living organisms. Structurally, it is a linear polysaccharide consisting of N-acetyl-d-glucosamine and glucuronic acid. Hyaluronic acid interacts with receptors found on the surface of cells, thus promoting wound repair processes [125,126,127]. In the wound healing process, high molecular weight hyaluronic acid is produced by platelets which further stimulates fibrinogen deposition and, as a major component of the edema fluid, it promotes neutrophils recruitment for debris removal and tumor necrosis factor-α and interleukin-1β and -8 release. Subsequently, as it is fragmented to low molecular weight hyaluronic acid which will bind to CD44 receptors, leucocytes and monocytes are recruited. Moreover, it will interact with toll-like receptor-2 and -4 present on lymphocytes and macrophages. Finally, hyaluronic acid guides fibroblast invasion and proliferation, which is fundamental for collagen deposition, and differentiation into myofibroblasts for wound contraction [128]. Therefore, hyaluronic acid-based hydrogels have been widely investigated for wound healing purposes, with the most common comprising glycidyl methacrylate-, thiol-, and DNA-functionalized hyaluronic acid [127].

Collagen, another extracellular matrix component, is the most commonly found protein in the body produced by fibroblasts and involved in cellular and molecular cascades involved in wound healing and debridement and tissue regeneration [127]. Collagen dressings are capable of counterbalancing the elevated levels of matrix metalloproteinases which are usually released at the wound site and proteolytically deteriorate native intact and partially degraded fragments of collagen molecules. Currently, collagen is coupled with other natural and synthetic polymers, e.g., salmon milt DNA, anionic polysaccharides, minocycline based hydrogels, α-tocopherulate, and alginic acid, for developing novel dually functional collagen-based wound dressings that combine both wound healing and exudate absorbing properties [127,129,130].

Alginate is natural anionic polymer extracted from brown seaweed widely applied in the biomedical field owing to its biocompatibility, non-toxicity, and low cost [127,131]. Structurally, alginate is linear branchless polysaccharide consisting of various (1→4′)-linked β-D-mannuronic acid and α-L-guluronic acid subunit contents [127]. It is widely used as a wound dressing biomaterial as it fulfills the requirements regarding exudate absorption and tissue regeneration. Specifically, owing to its hydrophilic nature, alginate is able to absorb high amounts of wound exudate, while also maintaining the required moisture and exhibiting a hemostatic effect [132,133]. Additionally, it enhances cell migration, increases angiogenesis, promotes collagen type I production, and suppresses pro-inflammatory cytokine concentrations for skin regeneration and prevents bacterial contamination within the wound site [127,132]. Alginate can be easily crosslinked with other organic or inorganic materials, such as calcium, sodium, collagen, and gelatin [127,133], which is reflected by the large number of commercially available alginate-based dressings [132].

However, although they are highly efficient, interactive wound dressings are unable to solve deeper lesions or effectively prevent microbial infections. Therefore, bioactive wound dressings are more promising as they promote healing processes by the gradual release of the biocompounds encapsulated within [25,134,135,136]. Moreover, for skin cancer applications, the regenerative properties of wound dressings are not sufficient as they require the presence of anti-cancer agents for preventing cancer recurrence. Therefore, wound dressings consisting of biopolymers as the regenerative component and natural anti-cancer agents as the cancer recurrence-preventing component could represent ideal candidates to use for regenerative applications in skin cancer.

In this context, Shukla et al. [137] developed hydrogels consisting of gellan gum and chitosan crosslinked with poly(ethylene glycol) loaded with apigenin. Their results on rat wound models showed a 96.11% release of the bioactive compound within 24 h and significant antioxidant activity. While the unique properties of the hydrogels in terms of biocompatibility, biodegradability, moisture, and antioxidant efficiency are considerably promising for wound healing and regeneration, the release of the bioactive compound should be gradual in order to ensure an optimal concentration at the wound site for longer periods.

Moreover, quercetin-impregnated chitosan and fibrin scaffolds were developed by Karthick et al. and Vedakumari et al., which exhibited ideal mechanical strength for a wound dressing material, in vitro non-toxicity and bactericidal effects against *Escherichia coli* and *Staphylococcus aureus* strains, and accelerated wound healing after topical administration on albino rats [138]. George et al. investigated the therapeutic effects of a chitosan hydrogel crosslinked with dialdehyde cellulose containing phyto-derived quercetin extracted from onion peel waste and zinc oxide nanoparticles. The incorporation of nanoparticles increased drug loading within the hydrogel and inhibited *Staphylococcus aureus* and *Trichophyton rubrum* strains growth. In vitro tests showed good biocompatibility on normal L929 murine fibroblast cells and anti-cancer properties against A431 human skin carcinoma cell lines [139]. Furthermore, Jangde et al. [140] incorporated quercetin into a multiphase hydrogel consisting of Carbopol^®^ (the trade name for carbomers, high molecular weight cross-linked poly(acrylic acid) polymers) and varying gelatin ratio. The most suitable hydrogel was obtained at a ratio of gelatin to Carbopol^®^ of 6 to 4, which exhibited accelerated wound healing and reduced wound closure time albino rat models [141]. Ajmal et al. [142] designed a wound dressing based on poly(ε-caprolactone) nanofibers loaded with ciprofloxacin hydrochloride and quercetin in order to ensure both anti-bacterial and antioxidant properties. Results showed high entrapment efficiency of more than 92% for both biocompounds and a prolonged in vitro release for 7 days. Thus, the hydrogel was able to accelerate wound healing on full thickness wound models in rats by improving collagen deposition and re-epithelialization within 16 days and prevent reactive oxygen species production.

Silymarin was also evaluated as a bioactive compound for wound dressing application. In this regard, Tsai et al. [143] developed a bacterial cellulose nanofiber film onto which silymarin-loaded zein nanoparticles were adsorbed. Their findings proved enhanced antioxidant and anti-bacterial activities for silymarin-containing films, which protected salmon muscle against lipid oxidation and deterioration.

Additionally, Kim et al. developed crosslinked interpenetrating polymer networks hydrogels consisting of poly(N-isopropylacrylamide) and hyaluronic acid for the transdermal delivery of luteolin. Texture and rheometry analyses proved that the 3% crosslinker-containing hydrogel has the most adhesive and stable network, which was further applied for drug release evaluation. Results showed no cytotoxicity and inhibited keratinocyte hyperproliferation in psoriasis, which could further be applied in skin cancer applications in order to prevent cancer cell proliferation [144].

A novel fibrous material consisting of poly(ε-caprolactone) and chitosan containing ferulic acid was prepared by Yakub et al. through electrospinning or electrospinning combined with dip-coating. The composition and design of the matrix influenced ferulic acid incorporation and results showed higher anti-bacterial activity when compared to the application of ferulic acid-containing poly(ε-caprolactone) and chitosan-coated poly(ε-caprolactone) fiber mats against *Staphylococcus aureus* strains. Additionally, the incorporation of ferulic acid within the polymer matrix increased the anti-cancer character against HeLa tumor cells while maintaining its antioxidant activity [145]. Likewise, Poornima et al. investigated the release of quercetin and resveratrol from similar polymer matrices for their anti-inflammatory and pro-angiogenic activities, respectively. The in vitro studies showed a sustained release for both biocompounds of up to 48% and 55%, respectively, for 120 h. Furthermore, in vivo studies on rats resulted in complete wound healing in 15 days when treated with the biomaterials and 20 days for the control [146].

Moreover, caffeic acid has also been widely studied for its potential in regenerative wound dressing applications. In this context, Oh et al. [147] compared the effects of poly (ε-caprolactone), poly (ε-caprolactone) and chitosan, and poly (ε-caprolactone) and chitosan-caffeic acid conjugate fibrous mats fabricated by electrospinning. The chitosan-caffeic acid conjugate-based fibrous mat exhibited significantly increased tensile properties and higher initial cell attachment, cell proliferation, and anti-microbial effects, which proves its potential for wound healing and regeneration. Subsequently, Ignatova et al. designed and fabricated fibrous materials based on poly(3-hydroxybutyrate), quaternized chitosan, κ-carrageenan, and caffeic acid through electrospinning or electrospinning in conjunction with dip-coating and polyelectrolyte complex formation. Results showed that caffeic acid release is influenced by fiber composition. In this regard, caffeic acid-containing mats obtained through polyelectrolyte complex formation exhibited anti-bacterial character against *Staphylococcus aureus* and *Escherichia coli* strains and an enhanced antioxidant activity [148]. Similar results were obtained by the same research group for poly(3-hydroxybutyrate) and polyvinylpyrrolidone poly (3-hydroxybutyrate) containing caffeic acid phenethyl ester [149] and poly (ethylene glycol)-based fibrous materials containing caffeic acid [150].

Jaganathan et al. [151] prepared materials based on *Artocarpus heterophyllus*-derived lignin and chitosan with varying wt% concentrations for biomedical purposes. Lignin significantly improved mechanical stability of the material and biocompatibility towards NIH 3t3 cells, proving the suitability of the biocomposites for regenerative wound dressing applications. Moreover, Zhang et al. [152] introduced lignin into a polyvinyl alcohol and chitosan composite hydrogel for enhancing its mechanical strength and accelerate wound healing processes on murine wound models. Zmejkoski et al. [153] fabricated dressing materials based on bacterial cellulose and coniferyl alcohol composite hydrogel. Their findings showed a sustained release of the bioactive compound, with the highest release within the first hour and a slower release within the following 72 h, and inhibitory and/or bactericidal effects.

Resveratrol is another antioxidant biocompound extensively investigated for wound healing and regeneration. Therefore, Berce et al. [154] synthesized a polymeric sponge consisting of chitosan and sodium hyaluronate for the controlled release of resveratrol. In vitro and in vivo studies confirmed its potential to stimulate tissue regeneration by enhancing granulation formation which facilitates wound healing, while also achieving bacteriostatic effects. Similarly, Hussain et al. developed hyaluronic acid-functionalized chitosan nanoparticles for an efficient topical administration of resveratrol and curcumin. The optimized formulation was characterized by an entrapment efficiency of approximately 90% for both biocompounds and non-Fickian diffusion and sustained release mechanism for in vivo studies [155]. Furthermore, Gokce et al. [156] synthesized resveratrol-loaded hyaluronic acid and dipalmitoylphosphatidylcholine microparticles embedded within a dermal matrix consisting of collagen-laminin. The release of resveratrol was sustained, reaching 70% after 6 h. Application on full-thickness excision diabetic rat models resulted in improved collagen fibers as the addition of resveratrol delayed dermal matrix degradation by collagenases and prevented inflammation due to antioxidant properties. Lakshmanan et al. [157] developed electrospun poly(ε-caprolactone)-based scaffolds containing resveratrol. In vivo experiments full-thickness ischemic mice wound models showed a considerably faster wound closure and re-epithelialization than the collagen-treated and negative control groups. Additionally, the anti-apoptotic and regenerative potential was demonstrated through the activation of thioredoxin-1 and heme oxygenase-1 mediated vascular endothelial growth factor signaling and the expression of bcl-2 in wound edges after treatment.

Therefore, it can be concluded that the combination of biocompatible polymers which are known for both their regenerative and anti-microbial properties with natural anti-cancer agents is a promising strategy for the development of wound dressings that could be applied in skin cancer before or after surgical interventions. In this manner, these dressings could promote tumor regression and prevent cancer recurrence, without causing adverse effects as in the case of conventional topical field treatments. 

## 5. Conclusions and Future Perspectives

For skin cancer treatment, radical tumor excision remains the most effective approach among the available strategies. However, surgical interventions can be disfiguring, requiring additional skin grafts to cover the defects. In this context, post-surgery management should involve the application of wound dressings for promoting skin regeneration and preventing tumor recurrence and microbial infections, which still represents a considerable clinical challenge. While topical field administration is widely applied in skin cancer management, current options are still limited due to the various related side effects and the prolonged treatment periods. In this regard, plant-derived compounds have proved to exhibit DNA protection, antioxidant, anti-inflammatory, chemopreventive, and chemotherapeutic activities and could represent a promising alternative. Therefore, wound dressings consisting of biopolymers as the regenerative component and natural anti-cancer agents as the cancer recurrence-preventing component could represent ideal candidates to use for regenerative applications in skin cancer. As most studies focus on the healing potential, the number of publications on this subject is still relatively limited and further research is fundamental for developing regenerative wound dressings for skin cancer. In this context, research studies that not only assess the antioxidant potential of these bioactive dressings but also the anti-cancer properties should be performed.

## Figures and Tables

**Table 1:** Characteristics of the main topical field treatment options.

Agent	Chemical Formula	Commercial Formulation	Treatment Schedule	Side Effects
Imiquimod	1-Isobutyl-1*H*-imidazo(4,5-c)-quinolin-4-amine	3.75% cream (Zyclara) 5% cream (Aldara)	5–7 times per week once a day 6–12 weeks	Erosion, ulceration, healing with scarring or hyperpigmentation, erythema, vesiculation, edema, weeping, pruritus, scaling, crusting, burning, and pain
5-Fluorouracil	5-Fluoro-1*H*-pyrimidine-2,4-dione	2% solution 5% solution 5% cream (Efudix)	Twice daily 2 or more weeks	Allergic contact and irritant dermatitis, erythema, pruritus, erosions, hyper- or hypopigmentation, and pain
Ingenol mebutate	Ingenol-3-angelate	0.015% gel 0.05% gels	2 to 7 consecutive days	Erythema, edema, pruritus, and pain
Diclofenac	2-(2-(2,6-Dichloro-phenylamino) phenyl)acetic acid	3% gel	Twice daily for 8 weeks	Erythema, erosion, allergic contact dermatitis, photoallergy, and pruritus

**Table 2:** The main flavonoid compounds with anti-cancer potential for skin cancer treatment—chemical formula and structure, sources, and study references.

Compound	Chemical Formula	Chemical Structure	Sources	References
Apigenin	5,7-Dihydroxy-2-(4-hydroxyphenyl)-4*H*-chromen-4-one	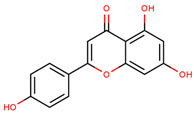	Parsley, celery, chamomile, oranges, onions, honey, thyme, oregano, rosemary, basil, coriander, tea, beer, and wine	[62,63,64,65]
Quercetin	2-(3,4-Dihydroxy-phenyl)-3,5,7-trihydroxy-4*H*-chromen-4-one	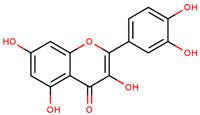	Apples, citrus, red onion, and the roots and leaves of many vegetables	[66,67,68,69,70,71]
Silymarin	3,5,7-Trihydroxy-2-[3-(4-hydroxy-3-methoxyphenyl)-2-(hydroxymethyl)-2,3-dihydro-1,4-benzodioxin-6-yl]-3,4-dihydro-2*H*-1-benzopyran-4-one	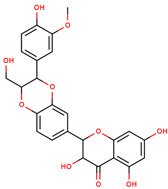	Milk thistle seeds	[72,73,74]
Diosmetin	5,7-Dihydroxy-2-(3-hydroxy-4-methoxyphenyl)-4*H*-chromen-4-one	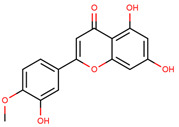	Rosemary, bergamot juice, and citrus juice	[75,76,77,78]
Genistein	5,7-Dihydroxy-3-(4-hydroxyphenyl)-4*H*-chromen-4-one	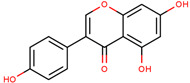	Soy	[79,80,81,82]
Fisetin	2-(3,4-Dihydroxy-phenyl)-3,7-dihydroxy-4*H*-chromen-4-one	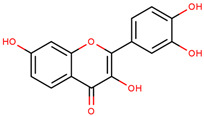	Strawberries, apples, persimmons, grapes, onions, kiwi, and kale	[82,83,84]
Luteolin	2-(3,4-Dihydroxy-phenyl)-5,7-dihydroxy-4*H*-chromen-4-one	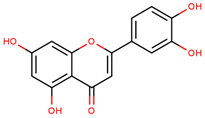	Carrots, peppers, celery, olive oil, peppermint, thyme, rosemary, and oregano	[85,86]

**Table 3:** The main phenolic acids with anti-cancer potential for skin cancer treatment – chemical formula and structure, sources, and study references.

Compound	Chemical Formula	Chemical Structure	Sources	References
Syringic acid	4-Hydroxy-3,5-dimethoxybenzoic acid	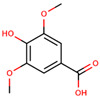	Olives, dates, spices, pumpkin, grapes, acai palm, honey, and red wine	[90,91,96]
Ferulic acid	3-(3-Hydroxy-4-methoxy-phenyl)prop-2-enoic acid	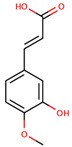	Whole grains, spinach, parsley, grapes, rhubarb, and cereal seeds, mainly wheat, oats, rye, and barley	[92,97]
Caffeic acid	3-(3,4-Dihydroxy-phenyl)prop-2-enoic acid	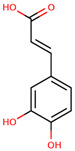	Coffee, wine, tea, and propolis	[93,94,95,98]

**Table 4:** The main stilbenes with anti-cancer potential for skin cancer treatment—chemical formula and structure, sources, and study references.

Compound	Chemical Formula	Chemical Structure	Sources	References
Resveratrol	5-[(*E*)-2-(4-Hydroxy-phenyl)ethenyl] benzene-1,3-diol	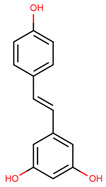	Grapes, wine, nuts, and berries	[108,109,110]
Pterostilbene	4-[(*E*)-2-(3,5-Dimethoxy-phenyl)ethenyl] phenol	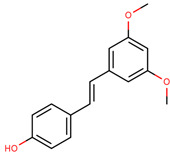	Grapes, wine, nuts, and berries	[111,112]
Piceatannol	5-[(*E*)-2-(3,4-Dihydroxy-phenyl)ethenyl] benzene-1,3-diol	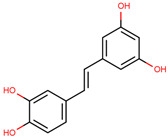	Grapes, berries, and red wine	[113,114]
